# Factors influencing intention to participate in breast cancer screening. An exploratory structural model

**DOI:** 10.1371/journal.pone.0281454

**Published:** 2023-02-03

**Authors:** María Belén López-Panisello, María José Pérez-Lacasta, Montserrat Rué, Misericòrdia Carles-Lavila

**Affiliations:** 1 Department of Business Management, Rovira i Virgili University, Reus, Spain; 2 Research Group on Statistics, Economic Evaluation and Health (GRAEES), Reus, Spain; 3 Department of Economics, Rovira i Virgili University, Reus, Spain; 4 Research Centre on Economics and Sustainability (ECO-SOS), Reus, Spain; 5 Department of Basic Medical Sciences, University of Lleida, Lleida, Spain; 6 Lleida Biomedical Research Institute (IRBLLEIDA), Lleida, Spain; 7 Health Services Research on Chronic Patients Network (REDISSEC), Madrid, Spain; Lorestan University, ISLAMIC REPUBLIC OF IRAN

## Abstract

**Objectives:**

The paper has two objectives. The first one examines whether informing women about the benefits and adverse effects of breast cancer screening could have an effect on three variables: their knowledge, the importance women attach to the future consequences of their current decisions (time perspective), and the degree to which women are worried about developing breast cancer (worry). The second one examines whether these three variables affect their intention to participate in the screening, either directly or indirectly through their feeling of regret if they do not attend the screening (anticipated regret); through their values and the support they receive in making their decisions (decisional conflict); and, through the perceived acceptability and benefits of the screening programme (attitude).

**Methods:**

Partial least squares-structural equation modelling (PLS-SEM) is used to analyse both objectives and to differentiate between direct, indirect, and moderating effects, due to the incorporation in the model of the three mediating variables (anticipated regret, decisional conflict, and attitude) and a moderating variable (educational level).

**Results:**

Information affects knowledge (objective variable), but not the behavioural variables (time perspective and worry). On the other hand, the level of knowledge has no direct or indirect effect on intention, but behavioural variables do affect it through the mediating variables.

**Conclusions:**

The variables of the planned behaviour theory are relevant to understand women’s decisions and to be able to take appropriate health policy measures. Doing so, the processes of personalised screening would improve, or there would be the incorporation of shared decision-making in this context; these being demands associated with the most recent goals achieved in health programmes in many countries.

## 1. Introduction

Breast cancer (BC) is the most common cancer among women and, the second leading cause of cancer mortality in developed countries (15.4%) after lung cancer [1]. The mammography enables to detect cancer at early stages, reduce breast cancer mortality by 20% and prevent one death out of 235 women invited to undergo screening for 20 years [2]. However, the mammography has also adverse effects (overdiagnosis, false positives and false negatives), which introduce a trade-off between benefits and harms in the opportunity to participate [3]. When this uncertainty is due to the coexistence of both benefits and harms, women must be provided with information so that they can make an informed decision. A decision which will often depend not only on the received information, but on their social context, values, and preferences as well.

Informing women about the benefits and adverse effects of breast cancer screening could have an effect on their knowledge; their time perspective or their worry about breast cancer, and also affect their intention to participate in the screening, directly or indirectly, through changes in anticipated regret, decisional conflict or attitude towards the screening, as it has already been analysed in many studies [4–8].

This article aims to explore cause and effect relationships between these variables.

The database we have used was collected in a previous study. The InforMa study [9] conducted a controlled randomised trial to assess the impact of using a Decision Making Tool on informed decision making in breast cancer screening within four population-based programmes in Spain. Initially, women were seen to have no knowledge of either the benefits or harms of mammography screening. Yet, when the intervention group received the information, both knowledge and informed decision making increased considerably (i.e., the result of having proper knowledge on benefits and harms together with consistent attitudes was this: a positive attitude and willingness to participate or, a negative attitude and unwillingness to participate); on the contrary, decisional conflict decreased (i.e. the uncertainty of choosing when alternatives have various risks or do not wholly agree in reference to personal values). Nevertheless, there were no differences between both groups regarding either their attitude or intention to participate in the screening programme.

Regarding methodology, structural models are useful to understand how and why these results occur. They complement the direct analysis that comes from comparing both explanatory and explained variables, when adding indirect effects which can appear through a set of mediating variables. The paths constructed through these mediating variables constitute a set of indirect relationships that can either reinforce or go in the opposite direction, or even cancel out the direct effect.

The theoretical basis for the construction of the model proposed in this paper consists of social knowledge patterns and health behaviour [10]. The theory of Reasoned Action, based on consumers’ rational behaviour, can help understand the way the intention to participate or not in the screening is constructed. Briefly, the Reasoned Action model establishes that the intention to behave one way or the other responds to a balance between what someone believes one has to do (one’s own attitudes), and someone’s perception of what others believe one should do (subjective norm).

The theory of Planned Behaviour [11] widens the previous one, adding the consumer’s perception of factors which can facilitate or impede carrying out her decision (perceived behavioural control) and her own capacity to carry it out (real behavioural control), as explanatory factors of intention. At the same time, both attitudes and behavioural control can be either individually or socially based or be built upon the basis of the information received. In a screening context, there is evidence that the theory of planned behaviour variables is associated with mammography intentions [12].

In a recent analysis, Hersch [13] applied a causal inference model on the basis of the results of a controlled randomised trial in which the intervention consisted of providing information on both benefits and harms of breast cancer screening. Starting from a single independent variable, i.e., the provision of information on overdiagnosis, she constructs a pathway to learn about the causal chain through the variables: Over-detection Knowledge, BC Worry-Screening Attitudes, Anticipated Regret (mediating variables), which allow her to verify the reason why informed women were more unwilling to participate in the screening, which was the result obtained in her trial.

In contrast, our model incorporates three explanatory variables: knowledge about the benefits and harms of screening, plus two others from the theory of Planned Behaviour and the theory of Reasoned Action: BC worry and time perspective. Some studies have established the influence of CM worry [6] or time perspective [5] on the intention to participate in the screening. Moreover, we also incorporate the possibility of each of these variables by explaining the intention to participate in the screening directly or indirectly, through the effect they may have via mediating variables already identified in other studies: anticipated regret [7, 8], decisional conflict [14] and attitude towards the screening [12].

We have built a structural model to analyse the effect of the information provided by an aid-decision tool (AIDs) on women’s level of knowledge, worry and time perspective towards breast cancer. These three variables combine individual-social, behavioural-objective perspective, and they are considered the explanatory variables in the model. In order to analyse the intention, as the explained variable, we will conform various pathways that will let us analyse the indirect effects through the mediating variables: Regret, Conflict, Attitude and the moderating variable, Educational Level. Finally, we are introducing two control variables, Attitude and Intention (see Table 1).

**Table 1:** Variables of the model.

Exogenous explanatory variable	Endogenous explanatory variables	Moderating variable	Mediating variables	Explained variable	Control variables
Information about benefits and harms of breast cancer screening	Knowledge: the sum of the conceptual and numerical knowledge ratings about mortality reduction, false positives and overdiagnosis	Educational Level	Regret: how regretful women would feel if they had not attended screening	Intention: having no intention to participate (or being doubtful) or having it	Attitude Intention
			Conflict: certainty, knowledge, values and support received in making their decisions		
	BC Worry: the degree to which women report being worried about developing breast cancer				
			Attitude: acceptability and perceived benefits of the screening programme		
	Time Perspective: the importance of future consequences of women’s current decisions				

Consequently, this paper aims at building a structural model with mediating and moderating variables in order to analyse how women’s knowledge level and their individual and social beliefs may all influence on their intention to participate in breast cancer screening.

## 2. Materials and methods

### 2.1. Sample and procedures

The sample used comes from the InforMa project, a two-stage randomised controlled trial. At the first stage, 40 elementary territorial units of the Spanish public healthcare system were selected and randomised for intervention or control. At the second stage, 400 women aged between 49 and 50 were randomly selected and assigned to two different groups: control and intervention. The inclusion criteria were the following: they were women, aged 49 and 50 years, and the fact that in 2–4 months’ time they would be invited to participate in the screening programme for the first time. The women excluded were those who had a personal history of breast cancer; difficulty in speaking Spanish or Catalan, or a cognitive impairment that prevented them from understanding or completing the materials. Participants came from: Hospital del Mar in Barcelona, Cancer Prevention and Control Programme of the Catalan Institute of Oncology, the Canary Islands Health Service, and Lleida Health Region. Then they had to answer two questionnaires, at pre and post intervention. The intervention consisted of providing a decision-making tool with detailed information on the benefits and harms of breast cancer screening. The study analysed whether receiving this information affected women’s level of knowledge, their time perspective, and their worry about Breast Cancer. Participants were stratified according to their socioeconomic status and, all of them were at the stage of being called for the first time to participate in the breast cancer screening programme (49-50-year-old women). Women in both study groups were similar as regards to their sociodemographic variables, family history of breast cancer, perceived knowledge on benefits and harms of breast cancer screening and opinions on breast screening participation. More than 85% of the women in both groups considered that screening participation is important or very important. The complete demographic and health characteristics of those women can be found in the published article [9].

The study was approved by the Ethics Committees of Arnau de Vilanova University Hospital in Lleida (approval number 19/2014), Parc de Salut Mar in Barcelona (2014/5998/I), Bellvitge University Hospital in Hospitalet (PR349/14), and by the Scientific and Ethics Committee of Nuestra Señora de la Candelaria University Hospital in Tenerife (Canary Islands, Spain). Women who met the inclusion criteria and had agreed to participate were asked for informed consent, which was orally recorded.

### 2.2. Measures

Information is the exogenous variable of the model, and it is incorporated as a dichotomous variable (yes/no), reflecting membership in one or the other group of the InforMa study.

Knowledge, time perspective and worry are latent endogenous variables that can influence on intention to participate in the screening programme, directly and/or indirectly. The first one refers to the level of knowledge, measured as the sum of the conceptual and numerical knowledge ratings about mortality reduction, false positives and overdiagnosis, according to Hersch et al.’s study [15]. Time Perspective is assessed through the sum of four items referring to the importance of future consequences of women’s current decisions [16, 17]. Worry is measured by a single item which captures the degree to which women report being worried about their developing of breast cancer [18, 19].

Anticipated regret, decisional conflict and attitude towards screening are the mediating variables of the model which are used to search for indirect relationships between knowledge, time perspective and worry for intention. Anticipated regret is measured by an item which asks about how regretful women would feel if they had not attended screening [20, 21]. Decisional conflict is measured through a ten-item scale divided into four subscales: certainty, knowledge, values and the support received in their decision-making [22]. Attitude is measured by an adapted four-item scale which include acceptability and perceived benefits of the screening programme [23].

On the other hand, the intention to participate in the screening in the next 2 or 3 years is the main latent variable explained in the model. It is measured by a single item, which asks directly about these intentions and whose response can be categorised as having no intention to participate (or being doubtful) or having it [24, 25].

Finally, the model also contains two control variables which include both the attitude and intention expressed by women before being given information, in order to avoid getting distorted results.

The questionnaires carried out are published as Additional files 3 and 4 in https://doi.org/10.1186/s13063-017-2161-7 [26].

The database we have worked with can be found in the public repository: https://doi.org/10.34810/data214, which includes an explanation of how the variables are defined and the way they have been measured.

### 2.3. Research hypothesis

**Fig 1:**
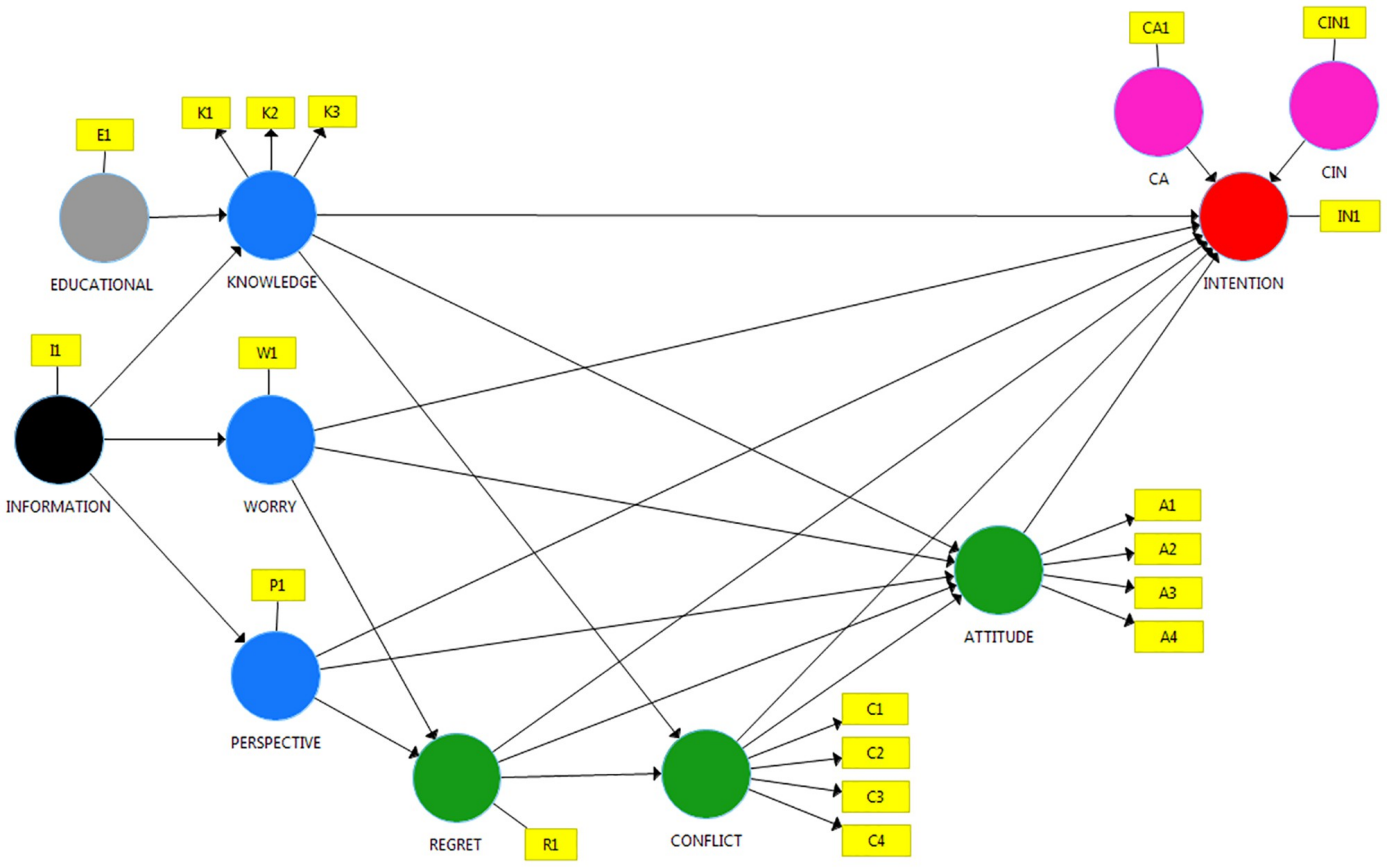
Conceptual model. I1: Information received by women. E1: Educational level. K1: Mortality reduction; K2: False positives; K3: Overdiagnosis. W1: Worry. P1: Time perspective. R1: Anticipated regret. C1: Certainty; C2: Knowledge; C3: Values; C4: Support. A1: Benefits of screening; A2: Adverse effects of screening; A3: Goodness of screening option; A4: Importance of screening. IN1: Intention to participate in the screening. CA1: Attitude before receiving information. CIN1: Intention before receiving information.

The conceptual model and the hypotheses we propose are shown in Fig 1. Based on the little existing theory discussed in the introduction of this paper, we define three types of hypotheses to make an exploratory analysis: direct effects, indirect effects, and a moderating effect.

Specifically, the first pathway emerges from the variable concerning the information received by women, which should influence on their level of knowledge; yet, with an intensity moderated by the variable concerning their educational level, since information improves knowledge to a greater extent for people with a higher level of education [27]. In turn, the level of knowledge is related to women’s intention to participate in the screening either directly or indirectly; even though, only the indirect way is expected to be significant [9]. The indirect relationship would occur through attitude and decisional conflict, either by parallel or sequential mediation. Anticipated regret is not included as a mediating variable since it is considered not to be affected by the level of knowledge, even though it is affected by behavioural variables.

The two other pathways explore the effect of the information received on BC worry and time perspective. Nevertheless, both variables are expected to be independent from the information received, since they are behavioural variables. These two variables could influence indirectly, either in a parallel way or sequentially on the participating intention through anticipated regret, decisional conflict, and attitude or, directly.

### 2.4. Statistical analysis

The statistical analysis to test our model and hypothesis was conducted by using the Partial least squares-structural equation modelling (PLS-SEM) technique and the statistical package Smart-PLS (v. 3.2.8) [28]. The main advantages of applying this technique to our research are the following: (1) it enables to effectively perform predictive and exploratory analyses of complex problems on which there is little theoretical knowledge [29–31]; (2) it allows the use of data which do not follow a normal distribution and (3) it enables the modelling of unobservable variables measured by one or several indicators [32].

Before evaluating the PLS-SEM results, it is important to remark that the statistical power of the sample [33] is good (0.917), because for a 400-women sample, an effect size of 0.15 and an error of 0.05, it is above the minimum value of 0.8, established by Cohen [34]. On the other hand, the approximate fit of the model is also adequate, since the Standardised Root Mean Square Residual (SRMR) indicator [35] presents a value of 0.086, lower than the limit value of 0.1, indicated by several authors [36, 37].

## 3. Results

Following Hair et al. [38], the evaluation of the results obtained for the proposed research model started describing and assessing the measurement models. Then, after verifying that this evaluation was satisfactory, the structural model was also evaluated.

### 3.1. Measurement model assessment

Reflective measurement models were assessed using individual item reliability, construct reliability, convergent validity and discriminant validity [39] (see Tables 2 and 3). Firstly, the individual item reliability was determined by examining indicator loadings. Results showed that all outer loadings, except that of item C2, are over the recommended cut-off value of 0.708 [32]. For the indicator C2 of the construct conflict, we follow Hair et al.’s recommendation [39]. They consider that if the indicator displays a value over 0.4, and in case of eliminating it, Composite Reliability or AVE do not improve; then, the indicator must be maintained (see Table 2). Secondly, the internal consistency reliability was tested using Dijkstra-Henseler’s Rho coefficient (*ρ* A) and Jöreskog’s composite reliability (CR) [26]. As shown in Table 2, all the constructs exceed the recommended cut-off value of 0.7 for the two coefficients [38]. Thirdly, the convergent validity of each construct was evaluated using the average variance extracted (AVE). Since AVE values are over the minimum value recommended of 0.5 [38], we have also proved the convergent validity (see Table 2).

**Table 2:** Individual reliability, construct reliability and convergent validity.

Construct /Indicator	Loadings	Dijkstra-Henseler’s Rho (*ρ* A)	Composite Reliability (CR)	Average Variance extracted (AVE)
Attitude (A):		0.916	0.938	0.791
• A1: Benefits from screening	0.862			
• A2: Harms from screening	0.915			
• A3: Goodness of screening option	0.939			
• A4: Importance of screening	0.837			
Conflict (C)		0.848	0.829	0.549
• C1: Certainty	0.836			
• C2: Knowledge	0.642			
• C3: Values	0.765			
• C4: Support	0.708			
Knowledge (K)		0.857	0.903	0.757
• K1: About mortality reduction	0.854			
• K2: About false positives	0.869			
• K3: About overdiagnosis	0.886			

**Table 3:** Discriminant validity.

	Ratio HTMT									
	A	R	CA	CIN	C	K	E	I	IN	P
R	0.482									
CA	0.196	0.144								
CIN	0.240	0.191	0.326							
C	0.330	0.373	0.063	0.118						
K	0.119	0.083	0.125	0.074	0.222					
E	0.037	0.035	0.079	0.077	0.100	0.134				
I	0.069	0.034	0.155	0.003	0.122	0.551	0.051			
IN	0.359	0.274	0.047	0.058	0.358	0.033	0.164	0.008		
P	0.263	0.282	0.220	0.212	0.223	0.039	0.022	0.040	0.178	
W	0.082	0.150	0.034	0.078	0.131	0.029	0.092	0.001	0.053	0.139

Note: Attitude (A); Anticipated Regret (R); Control Attitude (CA); Control Intention (CIN); Conflict (C); Knowledge (K); Education (E); Information (I); Intention (IN); Time Perspective (P); Worry (W).

Fourthly, discriminant validity was examined through the Heterotrait-Monotrait (HTMT) ratio, proposed by Henseler *et al*. [35]. Table 3 shows that discriminant validity is accepted because all constructs show a ratio lower than the recommended 0.85.

Once the reliability and validity evaluation were carried out, we concluded that our measurement models are completely satisfactory.

### 3.2. Structural model assessment

In order to determine the predictive relevance of the model, the values of the coefficient of determination (R^2^) and the Cross-validated redundancy (Q^2^) of all the endogenous, constructs were obtained. In turn, the bootstrapping statistical technique was used (5,000 subsamples) to establish the statistical significance of the direct effects, and also the empirical *t*-values and *p*-values were generated for all structural path coefficients. The bootstrapping technique was also applied to determine the significance of the indirect effects obtained in each path. Following Zhao’s procedure [40], the mediation analysis has been performed by testing the significance of these indirect effects. The moderation effect, however, was estimated using the two-step approach [41].

Before the hypothesis testing, collinearity between each set of predictor variables must be checked [32]. The Variance inflation factor (VIF) is frequently used to detect collinearity and, its value should be 3 or lower. The Smart-Pls results indicate that all VIF values are below 3, indicating the absence of collinearity among predictors.

Table 4 shows the results of the predictive relevance model and the direct effects. Regarding the first question, all constructs present a determination coefficient (R^2^), higher or very close to 0.1, which is the recommended minimum value [42], except for, as expected, time perspective and worry; indicating this way, that the information has a low explanatory capacity for these variables. In addition to evaluating the R^2^ values, the Cohen *f*^2^ values were analysed. The effect size *f*^2^ allows assessing an exogenous construct’s contribution to an endogenous latent variable’s R^2^ value. The *f*^2^ values of 0.02, 0.15 and 0.35 indicate an exogenous construct’s small, medium, or large effect, respectively, on an endogenous construct. The effect size of information on knowledge is large and regret has a medium effect on the construct conflict. The size of the other effects is small. The construct perspective has a small effect size on regret and also the effect size of regret on conflict is small. Knowledge and regret have small effects on conflict and conflict has a small effect size on attitude and on intention. Finally, the effect size of attitude on intention is also small.

**Table 4:** Structural model results: Direct effects.

Direct effects	R^2^	Q^2^	Coefficient	*t*-Value^1^	*p*-Value	95% BCCI	*f* ^2^
Information->Knowledge	0.293	0.202	0.508***	14.926	0.000	[0.452; 0.564]	0.365
Information->Perspective	0.002	-0.003	0.040^ns^	0.819	0.207	[-0.041; 0.121]	0.002
Information->Worry	0.000	-0.008	0.001^ns^	0.016	0.494	[-0.081; 0.082]	0.000
Worry->Regret	0.092	0.070	0.113**	2.554	0.005	[0.038; 0.182]	0.014
Perspective->Regret			0.266***	4.469	0.000	[0.170; 0.366]	0.076
Knowledge->Conflict	0.162	0.076	-0.161***	3.285	0.001	[-0.240; -0.077]	0.031
Regret->Conflict			-0.382***	8.691	0.000	[-0.456; -0.313]	0.173
Knowledge->Attitude	0.270	0.194	-0.110**	2.819	0.002	[-0.173; -0.045]	0.016
Worry->Attitude			0.028^ns^	0.750	0.227	[-0.034; 0.091]	0.001
Perspective->Attitude			0.111*	2.206	0.014	[0.024; 0.192]	0.015
Regret->Attitude			0.340***	6.165	0.000	[0.249; 0.432]	0.124
Conflict->Attitude			-0.208***	3.397	0.000	[-0.314; -0.111]	0.048
Knowledge->Intention	0.190	0.143	-0.021^ns^	0.428	0.334	[-0.105; 0.061]	0.001
Worry->Intention			0.038^ns^	0.727	0.234	[-0.049; 0.124]	0.002
Perspective->Intention			0.058^ns^	1.181	0.119	[-0.021; 0.140]	0.003
Regret->Intention			0.065^ns^	1.272	0.102	[-0.015; 0.152]	0.004
Conflict->Intention			-0.249***	4.238	0.000	[-0.351; -0.155]	0.058
Attitude->Intention			0.217***	3.415	0.000	[0.107; 0.317]	0.041
Control Attitude->Intention			-0.029 ^ns^	0.543	0.294	[-0.114; 0.062]	0.001
Control Intention->Intention			-0.012^ns^	0.253	0.400	[-0.087; 0.062]	0.000

Notes: R^2^: Coefficient of determination; Q^2^: Cross-validated redundancy; BCCI: Bias corrected confidence interval.

^1^ 5000 bootstrap samples;

*p<0.05;

**p<0.01;

***p<0.001;

^ns^ Not significant (two-tailed test).

On the other hand, the results of the Cross-validated redundancy (Q^2^), obtained through the Blindfolding process, show that all endogenous constructs have predictive relevance, as their Q^2^ is over zero, except for time perspective and worry. For these two constructs, therefore, the out-of-sample predictive relevance is low.

#### 3.2.1. Direct effects

Direct effects capture the direct relationships between pairs of variables in the model. Receiving information on benefits and harms of screening, in this analysis, improves the level of knowledge (β = 0.508 and p = 0.000), but it does not affect time perspective or worry about breast cancer.

The knowledge level incidence on intention (β = -0.021 and p = 0.334), worry about breast cancer on intention (β = 0.038 and p = 0.234) and time perspective on intention (β = 0.058 and p = 0.119) indicate that none of them is significant. That is, there are no direct effects of either knowledge, worry or time perspective on intention.

Yet, there are significant direct relationships between some variables. Knowledge level significantly reduces decisional conflict (β = - 0.161 and p = 0.001) and also worsens women’s attitude towards screening (β = - 0.110 and p = 0.002); time perspective significantly increases anticipated regret (β = 0.266 and p = 0.000) and improves attitude (β = 0.111 and p = 0.014); and breast cancer worry increases regret (β = 0.113 and p = 0.005).

Sequentially, regret reduces the decisional conflict (β = - 0.382 and p = 0.000) and improves attitude (β = 0.340 and p = 0.000); and in turn, the greater the decisional conflict is, the worse the attitude and intention are (β = - 0.208 and p = 0.000) (β = - 0.249 and p = 0.000). Finally, the attitude reinforces the intention to participate in screening (β = 0.217 and p = 0.000). The results of direct effects are shown in Table 4.

#### 3.2.2. Indirect and interaction effects

The results of the indirect effects and the interaction effects are shown in Table 5. The indirect impact of the knowledge level on the intention to participate in BC screening is seen through three different ways, and all of them are significant: Knowledge-Conflict-Intention (β = 0.040, p = 0.008); Knowledge-Attitude-Intention (β = - 0.024, p = 0.016), and Knowledge-Conflict-Attitude-Intention (β = 0.007, p = 0.019). However, as they have an opposite sign, the total indirect effect of knowledge on intention cancels out and it is not significant (β = 0.024, p = 0.141).

**Table 5:** Structural model results: Indirect effects and interaction effects.

	Coefficient	*t*-Value^1^	*p*-Value	95% BCCI
Indirect effects				
Knowledge->Conflict->Intention	0.040**	2.393	0.008	[0.015; 0.070]
Knowledge->Attitude->Intention	-0.024*	2.148	0.016	[-0.043; -0.007]
Knowledge->Conflict->Attitude->Intention	0.007*	2.078	0.019	[0.002; 0.013]
Total indirect of Knowledge on Intention	0.024^ns^	1.075	0.141	[-0.011; 0.062]
Worry->Regret->Intention	0.007 ^ns^	1.116	0.132	[-0.002; 0.019]
Worry->Attitude->Intention	0.006 ^ns^	0.721	0.235	[-0.007; 0.021]
Worry->Regret->Attitude->Intention	0.008 *	1.781	0.037	[0.002; 0.017]
Worry->Regret->Conflict->Intention	0.011*	2.086	0.019	[0.003; 0.020]
Worry->Regret->Conflict->Attitude->Intention	0.002*	1.722	0.043	[0.000; 0.004]
Total indirect of Worry on Intention	0.034*	2.268	0.012	[0.011; 0.061]
Perspective->Regret->Intention	0.017 ^ns^	1.138	0.128	[-0.004; 0.045]
Perspective->Attitude->Intention	0.024*	1.820	0.034	[0.004; 0.047]
Perspective->Regret->Conflict->Intention	0.025**	2.941	0.002	[0.011; 0.046]
Perspective->Regret->Attitude->Intention	0.020*	2.117	0.034	[0.007; 0.036]
Perspective->Regret->Conflict->Attitude->Intention	0.005*	2.156	0.016	[0.002; 0.008]
Total indirect of Perspective on Intention	0.091***	3.433	0.000	[0.052; 0.137]
Interaction effect				
Information x educational level->Knowledge	0.146*	3.715	0.000	[0.012;0.171]

Note: BCCI: Bias corrected confidence interval.

^1^ 5000 bootstrap samples.

*p<0.05;

**p<0.01;

***p<0.001;

^ns^ Not significant (two-tailed test).

The indirect impact of worry about breast cancer on intention to participate in BC screening can be seen through three significant indirect pathways: Worry-Regret-Attitude-Intention, with a β = 0.008 (p = 0.037); Worry-Regret-Conflict-Intention, with a β = 0.011 (p = 0.019); and Worry-Regret-Conflict-Attitude-Intention, with a β = 0.002 (p = 0.043). The sum of all individual indirect effects (total indirect effect) is significant, with a coefficient of 0.034 (p = 0.012).

Finally, regarding the results of the indirect incidence of time perspective on the intention to participate in breast cancer screening, we have observed that four out of the five indirect paths analysed are significant: Perspective-Attitude-Intention (β = 0.024; p = 0.034), Perspective-Regret-Conflict-Intention (β = 0.025, p = 0.002), Perspective-Regret-Attitude-Intention (β = 0.020, p = 0.034), and Perspective-Regret-Conflict-Attitude-Intention (β = 0.005, p = 0.016). And as in the previous case, the total indirect effect is also significant (β = 0.091, p = 0.000).

Regarding education, the moderator variable of the relation between information and knowledge, its interaction effect was introduced in the model employing the two-stage approach, as it is the most versatile [32]. As it can be seen in Table 5, the educational level has a positive effect on knowledge (0.146) in addition to the direct effect of the information on knowledge shown in Table 4 (0.508). These results indicate that the relationship between information and knowledge is 0.508 for a mean level of education. For higher educational values (that is, if education is increased by one point of its standard deviation) the relationship between information and knowledge increases on the basis of the size of the interaction effect (that is, 0.508+0.146 = 0.654), while for lower values (that is, if education is reduced by one point of its standard deviation), the relationship between information and knowledge is reduced on the basis of the size of the foresaid effect (0.508–0.146 = 0.342).

**Fig 2:**
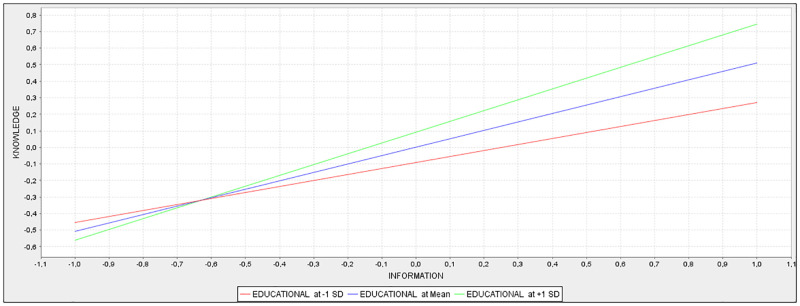
Simple slope analysis.

The simple slope graphic (Fig 2) was used to complete the analysis of the moderating effect. The graphic shows that there is a positive relationship between information and knowledge in the three lines since they all have a positive slope. The centre line represents the relationship between information and knowledge for a medium level of the education variable. The line at the top represents the relationship between both variables for high levels of education and, the one at the bottom represents the relationship between these variables for low levels of education. Therefore, higher levels of education lead to a stronger relationship between information and knowledge, since the upper line has a greater slope, the lower levels of education lead to a weaker relationship between information and knowledge, thus, the bottom line has a flatter slope.

In addition, the relationship between education and knowledge was determined to be significant, since the results obtained in the bootstrapping process with 5,000 subsamples provided a p value of 0.00. Therefore, we can affirm that the moderating effect of education on the relationship between information and knowledge is positive and significant, a result already obtained in other analyses [27].

## 4. Discussion

The results can be discussed at three different levels: firstly, on how providing information about benefits and harms of participating in breast cancer screening affects women’s level of knowledge, their level of worry about breast cancer and the time context in which they make their decisions. Secondly, which pathways are relevant to explain how these three variables affect the intention to attend, or not, the screening programme. Thirdly, the results provide insights into the way to reorient the organisation of screening programmes to take into account women’s preferences, and the values that inform their decision on whether to participate in the programme.

### 4.1. Effects of providing information on BC screening

Other studies [43, 44] have shown that providing users with balanced information does not affect their decision to participate or not in the screening programme, though it does reduce the hesitation to attend it or not [45].

However, this paper shows that it is necessary to disaggregate this result by separating the objective explanatory variables (knowledge) from those that depend on women’s behaviour (time perspective and worry). The inclusion of these last variables, not analysed until now, add a different perspective to the results found so far.

Thus, receiving information does not influence on either potential users’ time perspective of the BC screening programme or their worry about BC. Giving information to women significantly affects the objective level of knowledge that women subsequently demonstrate; in a more significant way, when their educational level is higher, since this variable considerably moderates this relationship.

This result confirms the hypothesis that time perspective and BC worry are related to behavioural variables, together with individual beliefs, preferences and values and social value factors, and therefore, they are not affected by the information provided by the decision-making supporting tool given to the intervention group.

BC worry is closely linked to each woman’s life experience (for example, if close friends or their relatives have suffered from the disease) to her personality, her general attitude towards the disease, her emotions, etc. The time perspective is a personal characteristic linked to one’s educational upbringing and the sociocultural background.

### 4.2. Relevant pathways on intention to participate in BC screening

In other articles [13] only knowledge was considered as an explanatory variable; no effects on intention to participate were found; it being confirmed when the analysis is conducted in the long term [46]. However, as it has been done in this paper, including behavioural variables, such as BC worry and time perspective, have defined new direct or indirect effects on intention to participate in the screening.

Referring to direct effects, no evidence has been found to stand up for a direct impact of the knowledge level, BC worry or time perspective on intention to participate in the BC screening programme.

On the contrary, some significant effects are obtained when the indirect effect analysis is introduced. A greater knowledge about benefits and harms of participating in the screening programme reduces decisional conflict, because it improves the knowledge about which options a woman has, when facing breast cancer screening, and which pros and cons there are for each choice.

A low-level decisional conflict positively affects the attitude towards screening, as women consider it important and worthwhile. Nevertheless, it surprisingly reduces their intention to participate, since women are probably more aware of its risks. These indirect pathways are contradictory, which makes the total indirect effect of knowledge on the intention to participate in screening almost non-existent.

Being worried about the possibility of developing BC increases anticipatory regret, which is measured as a woman’s fear: if she refuses to undergo screening when called, she will regret it later. The greater her fear is, the more worried she is about the possibility of developing the disease. Greater anticipated regret reduces decisional conflict (probably because it clarifies values, and she then better evaluates which pros and cons are more important for her if she undergoes it, or not) and that favours intention, as evidenced in other studies [47]. However, her level of worry does not significantly improve her attitude towards screening, because negative feelings may arise from the fact that screening tests may uncover a feared disease. In other words, anticipatory regret and decisional conflict are decisive mediating variables between women’s BC worry and her intention to participate in the screening programme.

A longer-term time perspective significantly increases anticipated regret, indicating the woman’s fear that, if she refuses to undergo screening when called, she will regret it later. Anticipated regret has a positive impact on attitude and intention. Greater anticipated regret highly reduces the decisional conflict and thus, influences on intention through attitude. Consequently, anticipated regret, decisional conflict and attitude towards screening are determining mediating variables between women’s time perspective and their intention to participate in the screening programme.

Therefore, only behavioural variables (worry about BC and time perspective) have an impact on their intention to participate in the screening programme, and it reveals that providing better information, which allows women to make an informed decision, does not affect their decision to participate in the programme.

### 4.3. Health policy implications for the organisation of screening programmes

Once it has been proved that behavioural variables are the ones able to influence the decision to participate in the screening, the current organisation of the programmes should be revised by introducing some key elements to learn about women’s preferences, beliefs and values and give them the possibility of making decisions consistent with them all.

In the 1990s Spain started some population screening programmes. At present there is a whole coverage: women between the ages of 50 and 69 can have a mammography every two years in public health centres.

Since up to now the mammography remains the best method for an early diagnosis, it is then advisable to continue participating in the programme. Just like most medical examinations, the mammography is uncertain and causes both adverse effects and benefits. However, as it is difficult to determine which women will suffer the harms or benefit from the screening, they should be advised on discussing their beliefs, preferences, and personal fears about screening with a healthcare professional and make a decision on the basis of informed and/or shared decision-making; a formula which could be introduced into current screening programmes. In addition, recent research has also shown that screening programmes could improve their effectiveness by changing from the current uniform age-based model to a personalised model including the individual’s risk of being diagnosed with breast cancer, her personal characteristics, her family history, age, genetic characteristics, previous history of breast disease, etc. In this way, the entry and exit age from the screening programme and the frequency and type of testing can be more precisely adjusted to each woman. Although there may be an initial resistance to change, this method would allow resources to be focused on women who require the greatest attention and, in conclusion, a more efficient use of resources would be achieved.

Both approaches, personalisation of screening and shared decision making, allow us to confirm a person-centred model and therefore, determine those behavioural variables we have identified as significant in our model for participation.

### 4.4. Strengths and weaknesses of the paper

This paper has focused on exploring an aspect which has been little studied so far in the field of health economics, that is, some behavioural variables effects on health outcomes such as the intention to participate in the BC screening programme. We have been able to demonstrate which pathways are relevant and which not, when understanding how women make their decision. We believe that one strength of this study is the introduction of these subjective variables (which basically depend on women’s preferences and values) to help better understand and explain this process and complement other previous studies which have not considered them.

In addition, the PLS methodology allows the introduction of non-directly observable variables but constructs, obtained through the measurement of several indicators. This allows using variables, considered relevant for the analysis by several Behavioural Theories which, on many occasions, cannot be directly measured.

In terms of weaknesses, the available data for us have forced some constructs to be measured by only one indicator, which reduces their conceptual richness.

Moreover, since women’s decision making really involves several interrelated factors, carrying out an exhaustive exploration has been impossible, and we have discarded some relationships between variables in order to reduce its complexity. Although we have made the logic of this decision clear, there is always the risk of having reduced the coherence of the model.

Finally, the results may be sensitive to variations if the sample or the healthcare setting changes. Although some of our results are consistent with those obtained in other studies that possibility should be taken into account. The small number of conducted studies including behaviour explanatory variables for intention to participate in the screening prevent us from being able to test their generalisability in a robust way.

## 5. Conclusions

Behavioural variables influence on the way people make their decisions and should be included in analyses. Regarding the decision to participate in a BC screening programme, we have observed that informing women of both its benefits and its harms does not affect their intention to undergo the test; however, some personal features such as concern about this disease or having a longer time perspective do so. For this reason, health policy measures aiming at improving the efficiency of early detection programmes should consider the way to include these behavioural variables in their programming.

Although individuals’ subjective characteristics are not easy to be modified, knowing the ones that influence on the intention to participate in a screening programme is the first step to focus on the action objectives in order to achieve the programme efficiency.

The personalisation of the screening programme, according to the risk of suffering from this disease, is a claim increasingly supported by public academic health studies. Other more behavioural characteristics should be added to the objective characteristics which define the level of risk if the goal is maximizing the benefits of healthcare interventions on the individuals’ well-being and the efficiency of our proposals. Furthermore, there is a growing awareness that shared decision-making between patients and doctors on clinical decisions should be enhanced, as for instance introducing the possibility for women to discuss their preferences and fears with healthcare professionals, which can reduce their decisional conflict and improve their well-being.
